# 62 The Impact of ICD10 on the Incidence of Abuse in the National Burn Repository

**DOI:** 10.1093/jbcr/irac012.065

**Published:** 2022-03-23

**Authors:** Erica Hodgman, Kristin Wharton

**Affiliations:** Johns Hopkins Children's Center, Baltimore, Maryland; Johns Hopkins Children's Center, Baltimore, Maryland

## Abstract

**Introduction:**

Previously, cases of abuse may only have been coded as such when the investigation was complete, thus skewing the data available in the National Burn Repository (NBR) toward more severe or obvious cases of abuse. The introduction of the ICD10 coding system in 2015 brought with it the concept of “confirmed” and “suspected” cases of abuse. We hypothesized that the creation of these two categories might shift our understanding of non-accidental pediatric burn injuries.

**Methods:**

We queried the NBR, a retrospective database maintained by the American Burn Association (ABA) which contains data submitted by all ABA-verified burn centers as well as some non-verified centers, for all children under the age of 18. Data were cleaned using e-codes where appropriate. To create equal samples, we created two cohorts: the ICD-9 sample comprises all children treated from 2012-2014, and the ICD-10 sample includes all children treated from 2016 to 2018. Data from 2015 were omitted as the ICD-10 coding system was introduced part way through the year.

**Results:**

A total of 34,456 patients are included in the sample, including 18,783 (54.5%) in the ICD-9 group and 15,673 in the ICD-10 group. The most common causes of injury in both eras was scald (48.7% in ICD-9 and 55.2% in ICD-10), followed by fire/ flame (20.3% in ICD-9 and 20.9% in ICD-10). The overall rate of abuse (including both suspected and confirmed abuse in the ICD-10 group) was higher in the ICD-9 era than the ICD-10 era (4.2 vs 3.2%, p< 0.001). As seen in previous studies, children with injuries due to abuse were younger (median age 2.0 vs 3.0 years, p< 0.001), had a larger median burn size (3.0 vs 5.0%, p< 0.001), a longer hospital length of stay (2.0 vs 4.0 days, p< 0.001), and a higher rate of mortality (0.8 vs 0.5%, p< 0.001). In the ICD-10 cohort, only 17 cases were coded as “confirmed” abuse (0.1%) and 478 were coded as “suspected” (3.0%). In contrast, in the ICD-9 group, 789 patients (4.2%) were coded as being victims of abuse.

**Conclusions:**

We expected that adoption of ICD-10, with its ability to yield increased data granularity, would yield a higher overall incidence of injury. Specifically, we expected more patients with smaller injuries to be included in the new “suspected” category, given the lower threshold to apply that diagnosis. However, this was not demonstrated in the NBR dataset, and in fact the overall incidence of abuse decreased. Given the added layers of complexity within the ICD-10 system, it is possible that the differences reflect challenges in coding cases of abuse rather than actual epidemiological changes.

